# Preventing physical and emotional violence by teachers in public schools in Pakistan: study protocol for a cluster-randomized controlled trial of the intervention Interaction Competencies with Children – for Teachers (ICC-T)

**DOI:** 10.1186/s12889-025-23144-x

**Published:** 2025-05-24

**Authors:** Alaptagin Khan, Katharina Goessmann, Florian Scharpf, Fakhar Khawaja Saqlain Shah, Anette Kirika, Khadeeja Azhar, Abdul Wahab Yousafzai, Anke Hoeffler, Tobias Hecker

**Affiliations:** 1 Khyber Medical University, Peshawar, Pakistan; 2 Shifa International Hospitals, Islamabad, Pakistan; 3 McLean Hospital, Boston, USA; 4 Harvard Medical School, Boston, USA; 5 Institute of Interdisciplinary Research on Conflict & Violence, Bielefeld University, Bielefeld, Germany; 6 Clinical Psychology & Violence Research Group, Department of Psychology, Bielefeld University, Bielefeld, Germany; 7 Shifa Foundation, Islamabad, Pakistan; 8 Shifa College of Medicine, Shifa Tameer E Millat University Islamabad, Islamabad, Pakistan; 9 Department of Politics and Public Administration, University of Konstanz, Constance, Germany

**Keywords:** Children, Cluster-randomized controlled trial, Public schools, School violence, Teacher violence, Mental health, Functioning, Pakistan

## Abstract

**Background:**

Violence can have serious short- and long-term impacts on children and young people`s health, development and well-being. On a societal level, it represents a considerable obstacle to economic growth and advancement by lowering human and social capital. Since many children worldwide, especially in low- and middle-income countries, experience violence by teachers at school, there is an urgent need for efficacious and scalable interventions to prevent violence by teachers and improve teacher-student interactions. To this end, the present study examines the effectiveness of the intervention Interaction Competencies with Children – for Teachers (ICC-T) to reduce physical and emotional violence by teachers.

**Methods:**

The study adopts a cluster-randomized controlled trial design with schools (clusters) as level of randomization and three data assessment points: baseline assessment prior to the intervention, the first follow-up assessment 6 months after the intervention and the second follow-up assessment 18 months after the intervention. Across four different sites in Pakistan, a total number of 48 public single-gender high schools (24 girls’ and 24 boys’ schools) are randomly selected. After baseline assessment, half of the schools are randomly allocated to the intervention group, in which teachers at selected schools receive ICC-T, and the other half to the control group, in which teachers receive no intervention. At each school, 30 students in the sixth year of school and all teachers (expected average number: 15) are recruited. Thus, the final sample comprises at least 1300 students and 720 teachers. Data are collected through structured interviews, standardized cognitive tests and school records. Primary outcome measures are student- and teacher-reported physical and emotional violence by teachers in the past week. Secondary outcome measures include teachers’ attitudes towards violence and children’s mental health problems, quality of life and cognitive functioning. Other outcomes are students’ academic performance and peer violence. Data will be analyzed using multilevel analyses.

**Discussion:**

This study aims to provide initial evidence of the effects of ICC-T on children’s exposure to violence at schools, their well-being and functioning in the context of Pakistan.

**Trial registration:**

The clinical trial was registered at ClinicalTrials.gov (ClinicalTrials.gov, 2024) under the identifier NCT06001554 (Preventing Physical and Emotional Violence by Teachers in Public Schools in Pakistan (ICC-T_Pak), 2023) on August 21^st^, 2023.

## Background

Despite global efforts to outlaw violence against children, more than 1.7 billion children – 75% of all children worldwide – experience violence in their upbringing [1]. Strikingly, violence against children in various forms – physical, emotional, sexual or neglect—is most often perpetrated by adults in children’s close social circles, such as parents, caregivers, teachers and other authority figures, who should nurture and protect them [2]. The most common form of such violence is corporal punishment, defined as “any punishment in which physical force is used and intended to cause some degree of pain or discomfort, however light” [3]. Despite being more challenging to conceptualize and operationalize, emotional abuse, which may include spurning, rejecting or denying emotional responsiveness to a child, is a very common form of violence against children as well [4]. According to the World Health Organization, violence against children at the hands of teachers continues to be a serious issue, as 732 million minors aged 6–17 years are estimated to live in countries where corporal punishment in schools is not fully prohibited [5].

The potential negative impacts of childhood violence in schools are manifold and have been documented by numerous studies and reviews, including impairments of brain development, cognitive, emotional, and social functioning, and mental health [6–12]. A review by Gershoff et al. observed consistent links between physical punishment and detrimental outcomes for children across cultural, family and neighborhood contexts [13]. In line with this, Cuartas et al. [9] recently showed that children who were spanked exhibited greater activation in multiple regions of the medial and lateral prefrontal cortex, suggesting that the experience may alter neural responses to environmental threats in a manner similar to more severe forms of violence.

The majority of studies examining the nature and impact of violence against children comes from higher income countries [14] although approximately 90% of the children and adolescents in the world live in low- and middle-income countries (LMICs) [15]. While some evidence suggests that the effects of physical discipline on children’s adjustment may be moderated by the perceived normativeness of the practice in the specific cultural context [16], studies from different LMICs like Colombia, Ghana and South Africa indicate that children usually experience fear and distress in reaction to violence and process violent experiences as threatening [14, 17, 18]. Research shows there are no positive effects of physical punishment whatsoever on improving child behavior or other outcomes for children [13]. On the contrary, evidence suggests that children exposed to corporal punishment, irrespective of the socio-cultural context, are about 24% less likely to be developmentally on track than children who are not exposed to violence [14].

Like in many other LMICs, violence against children is widespread in Pakistan [19]. Corporal punishment of children in home and school settings has become part of a social norm as reflected in a number of survey reports. According to Plan Pakistan and Plan International, majorities of teachers and parents agree that teachers are justified in beating students who are rude or disobedient and that physical punishment is necessary for most children to ensure academic achievement [20, 21]. In line with such attitudes are reports that physical punishment was used in 89% of schools in Punjab, with the practice being most common in public schools, followed by private schools, and then schools for religious education [22]. Similarly, in a large study across 40 public schools in Sindh province, Khuwaja et al. [23] reported that overall 91.4% of boys and 60.9% of girls reported having experienced corporal punishment at school in the previous four weeks, even though the local government had banned corporal punishment in schools in 2011.

With more than 40% of the country’s population under the age of 15 [24], violence by teachers and its detrimental outcomes pose a significant threat to Pakistan’s social capital. Ahmad et al., [25] identified corporal punishment as the main reason for children leaving school at primary level in Pakistan. Given the strong association between exposure to violence and subsequent drug and alcohol use [26–29], violence at school is likely to contribute to the alarming levels of drug abuse observed in Pakistan [30]. As children who are victims of violence are commonly perpetrators of violence against family and community members in later life [31], childhood violence exposure is also likely to be a strong contributory factor to Pakistan’s extremely high prevalence rates of gender-based violence, with 70—90% of married women reporting abuse from their spouses at any time in their lives [32].

One way of enabling social change is through the law, e.g., legally protecting children from abuse with the expectation that legal reform will not only limit the use of these harmful practices but will also change attitudes and thus prevent violence. Socio-legal research from Sweden shows that legal bans on corporal punishment are closely associated with decreases in support and use of corporal punishment and can contribute to a societal change towards a violence free upbringing of children [33, 34]. In Pakistan, corporal punishment of children by teachers and caregivers in institutional settings and workplaces has been legally banned in the federal capital of Islamabad in 2021, but it is still not fully banned in the whole country and not in all settings, including families [35, 36].

While important, legal prohibition alone seems not sufficient to reduce violence against children, as indicated by findings of continuously high prevalence rates of corporal punishment at school in some regions despite existing legal bans [6, 34, 37]. Legal reforms need to be accompanied by programs that address deeply ingrained attitudes and social norms that endorse the use of violence as a necessary practice to raise and educate children. Research suggests that social norms shape beliefs and attitudes towards violent parenting, which in turn guide behaviors [16, 38]. However, this opens the opportunity of preventing violence against children by shifting attitudes and social norms that sustain it [39]. Furthermore, to transform a classroom culture that they themselves grew up with, teachers should be equipped with non-violent interactional skills and competencies as alternatives to violence.

### Evidence-based intervention programs to prevent violence by teachers

To date, there are only few evaluated interventions that have shown to be feasible and efficacious at reducing teachers’ use of violence against children in primary or secondary schools [40, 41]. One of them, the Good School Toolkit, an intervention that aims to shift school operational culture through individual behavior-change techniques, has been implemented and tested in primary schools in Uganda [42]. The IRIE Classroom Toolbox [43], another training program for teachers to support managing child behaviors and foster their social-emotional competence, has been implemented and tested in pre-schools and primary schools in Jamaica. More details on these programs have been discussed elsewhere [40].

The third intervention program in that line is Interaction Competencies with Children – for Teachers (ICC-T), which aims to reduce violence by teachers and improve student–teacher interactions using a week-long workshop format involving teachers. A unique aspect of ICC-T is that it explicitly addresses and seeks to change social norms and attitudes that perpetuate violent practices by encouraging teachers to question them in the light of their own personal experiences of violence. This creates the basis for teachers to be willing to learn, practice and apply alternative non-violent strategies (see more details below in the Methods section). ICC-T has been shown to successfully reduce physical and emotional violence by teachers in cluster-randomized controlled trials (cRCTs) in primary and secondary schools in Tanzania and Uganda [44–46]. In a trial in primary schools in Tanzania, the intervention also led to reduced rates of violence among peers. Currently, ICC-T is being evaluated further in ongoing cRCTs in Ghana, Tanzania, Uganda and Haiti [37, 47].

The present study will implement and evaluate ICC-T in a cRCT in Pakistan, for the first time in Asia and in a predominantly Islamic country. Given the differences between this context and previous implementations of ICC-T, and in line with recommendations for complex interventions [48], we first conducted a feasibility study with two schools in each of the planned four sites in Pakistan. The results of this unpublished study showed a high demand, acceptance and motivation regarding the intervention among participating teachers. Further, ICC-T could be feasibly implemented with few contextual adaptations and the uncontrolled design provided preliminary evidence that ICC-T can effectively reduce teachers’ use of and positive attitudes towards violent discipline in Pakistan. Based on these promising findings and the demonstrated feasibility and efficacy of ICC-T in other contexts, we proceed with testing the effectiveness of ICC-T in Pakistan in a definite cRCT [49]. So far, there have been no scientific effectiveness evaluations of interventions aiming to reduce teachers’ use of violence against children in the context of Pakistan, which underscores the need for this study. The only cRCT of a school violence program conducted in Pakistan, the Right to Play’s school-based intervention aiming to reduce peer violence in public schools, also found a reduction in student-reported corporal punishment by teachers [50]. However, this intervention did not explicitly target violence by teachers by reducing teachers’ positive attitudes towards violence and equipping them with non-violent alternative teaching strategies.

### Objectives and hypotheses

Following a similar approach as previous and ongoing cRCTs of ICC-T [37, 44–47], the present study aims to examine the effects of ICC-T on *a)* teachers’ use of physical and emotional violence against students, and *b)* on students’ functioning (mental health, well-being, and cognitive functioning) in secondary schools in different regions in Pakistan. Based on previous successes of ICC-T, we hypothesize that ICC-T will reduce teachers’ use of physical and emotional violence as reported by teachers themselves and by students in Pakistan schools (*primary outcomes*). Given ICC-T’s focus on changing teachers’ attitudes and the known negative effects of violence on children’s functioning, we also hypothesize that the intervention will reduce teachers’ positive attitudes towards violent discipline and improve students’ functioning as indicated by lower levels of mental health problems and higher levels of quality of life, and cognitive functioning (*secondary outcomes*). Similarly, we hypothesize that ICC-T will improve students’ academic performance and reduce levels of peer violence as reported by students (*other outcomes*).

## Methods

Our protocol adheres to SPIRIT guidelines (*Standard Protocol Items: Recommendations for Interventional Trials*).

### Study design

**Fig. 1:**
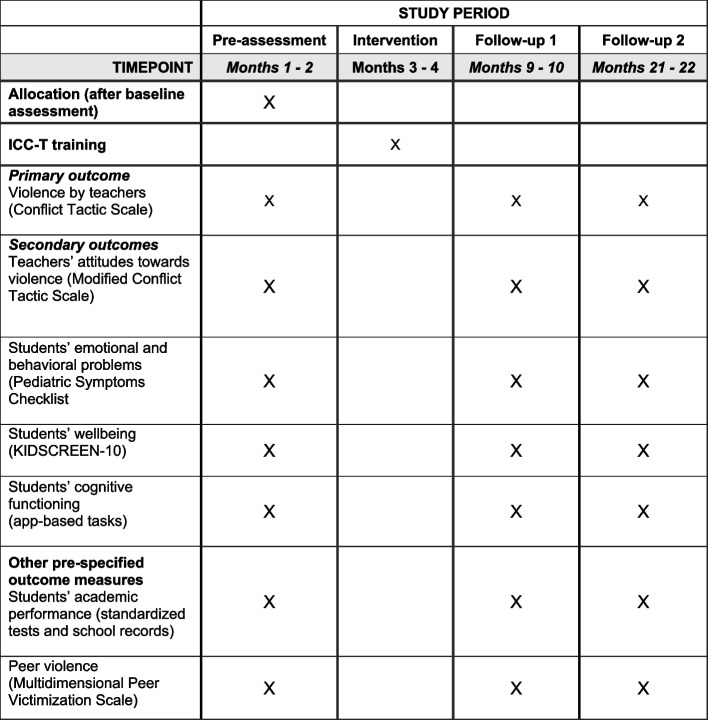
Study timeline

The study uses a two-arm multi-site cRCT design and is implemented in a total of 48 schools. Half of the schools are randomly allocated to the intervention group, which receives the ICC-T intervention, and the other half of schools to the control group, which receives no intervention. The study involves three data collection phases: baseline assessment directly before the intervention (t0), and two follow-up assessments approximately 6 months (t1) and 18 months (t2) after the intervention. Randomization of schools takes place after baseline assessment. See Fig. 1 for details.

#### Study setting and sampling

The study is carried out in four sites in Pakistan: the Islamabad Capital territory and the province of Punjab form one site and the other sites are the two provinces Sindh and Khyber Pakhtunkhwa and the administrative territory of Azad Jammu and Kashmir. These sites were chosen to consider different parts of the country that cover a broad range of geographical, cultural and socio-economic aspects. Within each site, three districts are purposefully selected to represent urban and rural areas. Within Islamabad Capital Territory and the province of Punjab, the three districts are the rural zone of Islamabad and the two Punjabi districts Rawalpindi and Attock. In Sindh, the three districts are Khairpur, Suukur and Naushoru Feroze. In Khyber Pakhtunkhwa, the three districts have been identified but remain to be finally confirmed due to a delayed start of the study activities. In Azad Jammu and Kashmir, the three selected districts are Bagh, Haveli Kahota and Poonch (Rawalkot). When this study protocol was submitted, baseline assessments and ICC-T workshops had already been started in all three districts in Sindh and in two districts in the combined site Islamabad/Punjab.

#### Schools

In each of the selected districts, schools meeting the following criteria are eligible for inclusion into the study. (1) Public, single-gender secondary/junior high schools. With public schooling being the primary model of education and physical punishment most common in public schools, our study is carried out in public secondary/junior high schools. Single-gender schools are selected because majority of the public schools in Pakistan, especially in the rural areas, are single-gender and the teachers are hired accordingly. (2) At least 30 students in the selected class. In case a selected class has fewer than 30 students, it is combined with a neighboring public single-gender school (within 15 km) to a school cluster and 15 students from each school are selected. (3) At least 15 and no more than 50 teachers at a school. In case of fewer than 15 teachers, a school cluster with a neighboring school (within 15 km) is formed and all teachers officially working at these schools are included. The upper limit of 50 teachers is due to practical difficulties related to providing the intervention to a high number of potential participants. (4) The school has not participated in a program aiming to reduce violence by teachers, improve teacher-student relationships and/or children’s wellbeing in the previous three years.

**Fig. 2:**
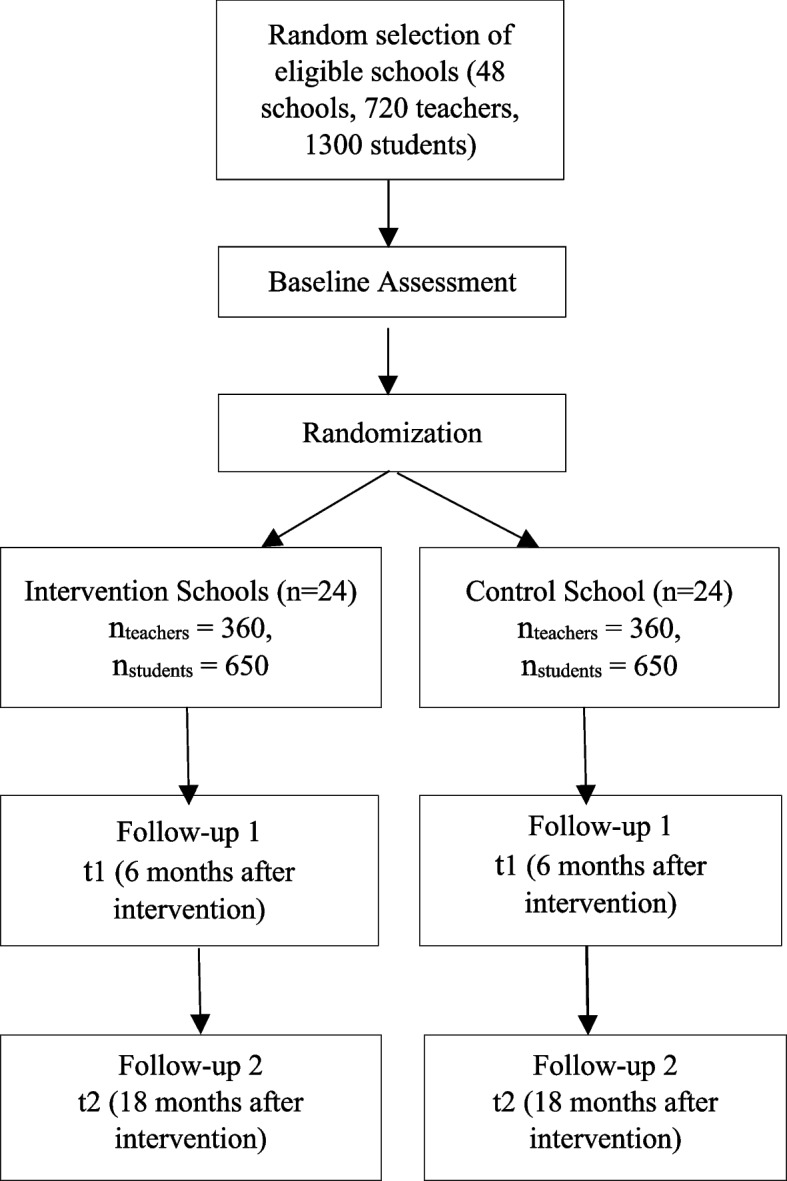
Sampling flow chart

Official lists of available schools are obtained from the relevant authorities and stratified by gender. After listing the gender-stratified schools in each selected district in alphabetical order, two boys’ schools and two girls’ schools are randomly selected, for a total of 12 schools per site (4 schools × 3 districts) and 48 schools in total. One school of the pair of single-gender schools in a district is randomly allocated to the intervention group, while the other school is allocated to the control group. This results in a total of 24 intervention schools and 24 control schools (six intervention schools and six control schools per site). A flow chart depicting the sampling procedures can be found in Fig. 2. All random selections of schools within districts as well as the allocation of schools to the two study conditions are performed by an independent researcher neither belonging to the core research nor the data collection teams using a random number generator.

#### Study participants

Due to the longitudinal nature of the study with three data collection points spanning across up to two years, secondary/junior high school students in grade 6 (approximated age: 12–14 years) are included in the study. Students must be below age 18 at the start of the study to be eligible for participation.

#### Sample size

The required sample size was calculated using the software *Optimal Design* [51]. Based on previous trials of ICC-T [37, 47], we expect moderate effects (δ = 0.25) for student-reported violence by teachers and large effects (δ = 0.35) for teacher-reported violence. In previous studies, the intra-class correlation was at 0.1 for teachers and 0.05 for students, and the covariance between baseline and follow-up scores of violence at the school level was around *r* = 0.30 [45, 46, 52]. We expect no drop out at the school level and an estimated attrition rate of 25% for students and 20% for teachers. Together with the specified parameters (α = 0.05; power = 0.80; effect size students: δ = 0.25, effect size teachers: δ = 0.35; ICC students = 0.05, ICC teacher = 0.01; R = 0.30) in total 48 schools with 27 students and 13 teachers per school are required to detect the expected intervention effects. The overall target sample is thus 1300 students and 720 teachers.

### Study procedures

Before data collection, local assessors and research assistants are selected and trained in the data collection procedures by the core research team in a one-week workshop. In addition to prior experience in research projects on social and health-related topics, the assessors are required to hold a university bachelor’s degree, be fluent in Urdu, which is the national language and the main mode of teaching across the country, as well as in the local language, and have basic level fluency in English. The assessors are blind to the allocation of the schools to the intervention and control groups. The assessment of students consists of a structured interview and a cognitive test, which take about one and a half hours in total. The assessment of teachers consists of a structured interview and takes about one hour. In the interview, assessors directly enter participants’ responses into Android tablets using the survey software SurveyToGo [53]. The cognitive testing is also administered to the students on the tablets using Inquisit Web [54]. All measures are administered with standardized introduction and administration procedures to ensure high objectivity and reliability during data assessment. All interview measures have been translated from English to Urdu according to recommended scientific guidelines including blind back-translation [55]. All interviews are conducted in Urdu to ensure participants’ full understanding. Recruitment of local bilingual assessors ensures that no information is lost in translation.

Prior to data collection, selected students receive a letter explaining the study aims and procedures together with an informed consent form for their parents to seek parental consent. Students whose parents have signed the informed consent form are invited to an interview in a quiet and discreet setting in the school premises. Before the interview, each student is given detailed written and oral information on the study procedure, the confidentiality of their data, and their right to withdraw from the study at any time without any consequences, and they are asked to provide their written consent to participation. Similarly, after being introduced to the study in a formal information session, all teachers at each selected school are invited to participate in an interview, and willing teachers receive detailed written and oral information on the study procedure, the confidentiality of their data, and their right to withdraw from the study at any time without any consequences, and provide written informed consent. The assessment procedures are repeated in the same way at 6-months and 18-months follow-ups. Students can be considered masked throughout the study as the intervention only targets teachers. Given the nature of the intervention, teachers are masked at baseline assessment but unmasked at the follow-up assessments.

#### Intervention description

The ICC-T intervention consists of a 5-day training workshop for teachers that is delivered by local facilitators and focuses on providing teachers with practical information and tools to prevent the use of violent disciplinary strategies in class and improve their interaction competencies with children. Sessions are divided into 5 modules: 1) Sessions about teacher-student interactions aim to promote teachers’ empathy and understanding of their students’ behavior and to raise teachers’ awareness of being a role model for students. 2) Sessions on maltreatment prevention aim to raise teachers’ awareness of the negative consequences of violent discipline on children’s well-being by inviting teachers to reflect on their own experiences of violence as a child and connect these experiences and associated feelings to the causes and consequences of their current violent behavior. 3) Sessions on effective discipline strategies aim to equip teachers with non-violent behavioral skills and tools helping them to maintain and reinforce desired behaviors and to change undesirable behavior by students. 4) Sessions on identifying and supporting burdened students intend to raise teachers’ awareness for common internalizing and externalizing problems among students and to increase their ability to identify and adequately support students with these problems. 5) Lastly, sessions on implementation aim at integrating the learned knowledge and skills into everyday school life and at ensuring sustainability to establishing support networks such as peer consultation and collaboration with school counselors (where available) and parents [56].

With behavior change as the primary objective, the content of ICC-T as an intervention was developed based on attachment theory, behavior modification theory, and social learning theory, and the central aspects of non-violent interaction between teachers and students being based on the work of the Democratic Classroom [57]^.^ For the implementation of the content, established methods that have been shown to lead to a change in behavior, such as inputs, role plays, discussion, and reflections, are used [56]. The structure of the training is important as it allows for a dynamic wherein a willingness to change is initially achieved, which is then followed by practical alternative strategies. Over the course of the week, an atmosphere develops in which curiosity, willingness to change, competence to act, and readiness to act (through motivation and social cohesion) catalyze each other.

#### Intervention procedures

The school-based ICC-T intervention is delivered by facilitators with a background in psychology and/or teaching who have participated in a two-week training including a pilot ICC-T workshop. Participation in the training workshop is free of charge. All teachers at a selected school receive detailed written information on the training procedure, the voluntary nature of their participation as well as their right to withdraw from the training at any point. Teachers who agree to participate in the training need to sign an additional informed consent form. In order to ensure that lessons can continue, half of the teachers in a school receive the intervention in one week and the other half of the teachers in the following week. Confidentiality of participants’ personal data and information shared during the training is ensured at any time. Treatment fidelity is assessed through questionnaires answered by the facilitators and randomly selected participants on each day of the workshop and through video recordings of pre-determined sequences of selected sessions. Questionnaires for facilitators cover information on each session’s duration, applied methods, deviations from the intervention manual and didactical aspects, as well as perceived uptake of the session content by participants. Questionnaires for participants focus on their perceived understanding of the day’s training content and the helpfulness of the applied methods in delivering the content. In addition, all participants evaluate the training contents and methods using a questionnaire at the end of the workshop. Video recordings are evaluated by independent raters to determine whether intervention workshops have been implemented in line with the manual. Contamination is assessed by interviewing the head teacher and five randomly selected teachers at each school around each follow-up timepoint about whether they shared materials or content from the ICC-T intervention with teachers in other schools.

#### Control condition

No intervention is implemented in control schools. The research team is in close contact with the control schools to closely document any similar intervention that takes place at the schools during the study. Apart from the intervention, all data collection procedures at baseline and follow-up assessments are implemented in control schools in the same way as in intervention schools. Similar to intervention schools, the head teacher and five randomly selected teachers at each school are interviewed around the follow-up timepoints about whether they had been in contact with teachers from other schools regarding student discipline.

### Outcomes

Primary, secondary and other outcomes are described below. All outcomes are assessed at all three assessment phases. The selection of assessment instruments considered both the psychometric quality and frequent use in comparable studies. The feasibility study of ICC-T in Pakistan demonstrated the applicability of the instruments in the context of Pakistan and no adaptations were made. Descriptive analyses will be used to examine basic psychometric properties of the measures in the present sample (see section Data analyses). The instruments are translated from English to Urdu using blind back-translation according to scientific standards [55].

#### Primary outcomes

##### Exposure to violence by teachers

Teachers’ use of physical and emotional violence against students in the past week is assessed using the Teacher–Child Conflict Tactics Scale (CTSTC), an adapted version of the Parent–Child Conflict Tactics Scale (CTSPC; [58]). The CTSTC is answered both by students as victims and by teachers as perpetrators of violence. It includes 16 items on experienced physical violence, seven items on experienced emotional violence and three items on witnessed violence by teachers, which are answered on a 6-point Likert scale from 0 (this has never happened) to 5 (more than 10 times). The items on experienced physical and emotional violence are combined to a total sum score as well as subscale scores. The CTS has been used in previous and ongoing trials of ICC-T and has demonstrated acceptable psychometric properties [37, 44, 45, 47, 52]. The CTSPC has been used in a national survey of violence against children in the home setting in Pakistan [59].

#### Secondary outcomes

##### Teachers’ attitudes towards violent discipline

An adapted version of the CTSTC serves as a measure of teachers’ favourable attitudes towards the use of physical and emotional violent discipline. Using the same 23 items as the CTSTC, teachers indicate to which extent they approve the respective act of violent discipline on a 4-point Likert scale from 0 (never OK) to 3 (always or almost always OK). The items are then summed up to a total score of attitudes towards violent discipline (range 0–69), with higher scores indicating more favourable attitudes. The scale has been used to assess teachers’ self-reported attitudes towards violent discipline in previous and ongoing trials of ICC-T [37, 44, 45, 47, 52].

##### Mental health problems

The 35-item version of the Pediatric Symptom Checklist – Youth Report (PSC-Y; [60]) is used to assess students’ emotional and behavioral problems in the past month. The items are rated on a 3-point Likert scale from 0 (*never*) to 2 (*often*) and summed up to a total score of emotional and behavioral problems (range 0–70). The PSC-Y has demonstrated good psychometric properties in a variety of cultural settings [61–63] and is being used in ongoing trials of ICC-T [37, 47]. The Urdu version of the PSC-Y has shown good reliability and validity at identifying adolescents with emotional and behavioral problems in public schools in Rawalpindi district [64].

##### Quality of life

The KIDSCREEN-10 [65] is used as a measure of children’s perceived quality of life. The 10 items cover physical, emotional, social, and behavioral aspects of well-being and functioning and are answered referring to the past week on a 5-point Likert scale ranging from 0 (*not at all)* to 5 (*extremely*). The KIDSCREEN-10 has demonstrated good psychometric quality in assessing children and adolescents’ self-reported quality of life in various cultural settings [65].

##### Cognitive functioning

Five tasks from the tablet application Inquisit are used to assess children’s cognitive abilities including selective attention (Letter Cancellation Task), working memory (Corsi Block Tapping Backwards), response inhibition (Arrow Flanker Task), planning and problem solving (Tower of London) and cognitive flexibility (Wisconsin Card Sorting Test).

#### Other outcomes

##### Academic performance

Students’ grades in core subjects (e.g. Mathematics, English, Science) in the last exams preceding the assessment are requested from the schools’ administrators and compared to exam results taken after the intervention.

##### Peer violence

The 24-item version of the Multidimensional Peer Victimization Scale [MPVS-24; 66] is used to assess children’s experiences of violence by peers in the past month. The MPVS-24 considers the six subtypes of physical victimization, verbal victimization, social manipulation, attacks on property, electronic victimization and social rebuff with four items each. The items are rated on a 3-point scale from 0 (not at all) to 2 (more than once) and combined into a sum score. The original 16-item and the 24-item version of the MPVS have shown good psychometric quality [66]. In addition, sexual violence by peers is assessed using four items from the adolescent version of the Sexual Experiences Survey [67] with the same response scale as the MPVS-24. A study with adolescents in Rawalpindi district found evidence of the reliability and validity of the 16-item version of the MPVS in the context of Pakistan [68].

### Efforts to reduce bias

The following efforts are expected to minimize the risk of bias and increase the validity of the findings. The random selection of schools within districts reduces selection bias. Comprehensive training of data collectors and the structured interview format aim at reducing participants’ reporting biases and at increasing the validity of responses. Allocation to intervention and control group at the cluster level performed by independent personnel following baseline assessment ensures that those collecting data are blind to the treatment conditions of the schools. While teachers’ reports of violence against students are a direct measure of behavioural change that considers the variety of daily interactions that teachers have with students, their reports could be influenced towards lower levels of violence by participation in the intervention. Students’ reports of violence by teachers therefore provide an additional valuable test of the intervention effect [42]. In addition, analyses will be carried out by an independent researcher not involved in field activities based on the groups as randomized (“intention to treat”) to minimize bias in statistical interpretation and reporting and to avoid incomplete accounting of participants and outcome events.

### Ethical considerations

As this research focuses on violence against children, ethical considerations and child protection are essential. The study has obtained ethics clearance from the Institutional Review Board and Ethics Committee of Shifa International Hospital (IRB # 040–23, approval date: April 28, 2023).

To protect participants’ identities, their data are pseudonymized through numeric codes. Participants’ data are stored only together with their individual code in a password-protected folder on a secure server. The document linking the numeric codes to individual participants is kept strictly confidential and separate from other data in a specific encrypted and password-protected file only accessible to selected members of the core research team. This also refers to video recordings of teachers participating in the intervention. Personal data are not disclosed to any other person without the participant’s permission or as required by the law. As behavioral intervention studies are minimum risk studies, no adverse events are expected as consequences of the intervention itself. However, the following may occur: first, questions about experiences of violence and mental health problems may evoke upsetting memories and feelings. Second, participants may present with severe mental health concerns, such as suicidal ideation or behaviour. Third, participants may experience severe ongoing abuse. In all cases, participants will receive immediate psychological support by a trained psychologist who is part of the research team. If necessary, appropriate referrals and follow-up for specialized services and further management are made on a case-by-case basis, with participant safety as the top priority. All adverse and unexpected events are documented by the research team.

### Data analyses

Analyses will be conducted using the statistical software *R* [69]. While an extensive evaluation of the psychometric properties of the study measures in the context of Pakistan is beyond the scope of this trial, we will examine internal consistency (through Cronbach’s Alpha) and criterion validity of the study measures (through bivariate correlations between theoretically related concepts) at each timepoint***.*** Baseline data will be used to examine the prevalence of maltreatment in different settings (across all study sites) as well as children’s mental health and well-being. The main analyses focus on testing the effects of the ICC-T intervention on primary (teacher-reported use of emotional and physical violence, student-reported experiences of physical and emotional violence), secondary (teachers’ attitudes towards violence, students’ mental health, well-being and cognitive functioning) and other outcomes (students’ academic performance and experiences of peer violence) at follow-up, with students and teachers as units of analysis. To test the effect of ICC-T over time, we will estimate linear mixed effects models including fixed effects of intervention group (ICC-T, control) and timepoint (baseline, follow-up 1, follow-up 2) and their interactions. Random effects for schools/school clusters and for participants (teachers, students) will account for the nested data structure and individual variability. Participant’ baseline score of the respective outcome and demographics (age and gender) will be included as covariates in the models. Missing data are only expected due to dropout at follow-up and handled through maximum likelihood estimation if the missing data are at least missing at random. This will be assumed if participants completing the follow-up assessments and those who are missing do not differ in primary outcomes and key demographic variables (age and gender) at baseline. Given the higher risk of type I errors associated with multiple outcomes, Holm-Bonferroni correction will be applied to primary outcomes and the false discovery rate (FDR) will be applied to secondary and other outcomes. Results will be presented including appropriate effect sizes and with a measure of precision (95% confidence intervals).

## Discussion

Since the experience of violence is closely related to psychological, educational, and social problems, this study contributes to strengthening the wellbeing of children and adolescents and to preventing mental health disorders, thereby potentially increasing human and social capital on a community and society level. Evidence from economics shows that education and health have a significant impact on socio-economic outcomes. An additional year of education raises an individual’s monthly income in Pakistan by about 7% [70]. Children who experience violence in school are more likely to drop out of school and are therefore more likely to live a life in poverty. This is critical for a young country like Pakistan where 0–14 years old children and adolescents make up 35% of the country’s population. Pakistan is at a critical juncture where lawmakers have finally realized that prevention of violence against children by teachers is not only necessary for the well-being of its youth but also for fostering socio-economic development in the long run. However, achieving this just through legal prohibition may not be enough. Teachers and other staff in institutional care settings need support and alternative behavioral strategies and tools.

Following the idea of a “bottom-up” approach towards the prevention of violence targeting schools as engines for societal change [34] this study’s implementation of ICC-T in Pakistan will expand the scientific evaluation of school-based interventions to reduce violence. ICC-T has been implemented successfully in Ghana, Uganda, Tanzania, and Haiti. The program is based on social learning and attachment theories and uses established methods for implementation of the content and achieving the goal of behavior change. Methods aiming to increase teachers’ empathy and change their attitudes are used as well as practicing of specific strategies to handle situations in the classroom non-violently.

### Limitations

Attitudes toward the use of violence are rooted in and intertwined with cultural and societal factors. The historic development and embedding of such attitudes and beliefs contribute to the fact that they cannot easily be eradicated. Hence, the expected changes in attitudes and behavior must be considered as preliminary only, as profound changes might take time. The duration of follow-up in this study might lead to underestimation of intervention effects, as the outcome measures will only capture changes within about 1.5 years. Some diffusion of intervention activities between control and intervention schools cannot be ruled out completely. Due to the random selection, it is possible that two schools allocated to control group and intervention group, respectively, are somehow close to each other, which might contribute to underestimation of intervention effects as well. We aim to assess potential diffusion of intervention contents by asking school principals and teachers about it. Furthermore, the longitudinal design of the study might provide challenges related to the retention of the sample. Several unforeseeable factors among participating children and teachers such as moving to another location, changing schools, dropping out of school or absenteeism might affect the findings of the study. Similarly, teachers’ schedules might interfere with training schedules, hence, flexibility in timing and providing the training workshops might be necessary. For a successful implementation of the study, it is further crucial to include relevant stakeholders of education and school administration on political and practical levels. However, participation is voluntary in our study, so we must consider the risk that a small number of schools willing to participate might affect the generalizability of the study results.

## Conclusion

This study is the first trial of a school-based approach aiming to explicitly prevent violence by teachers in Pakistan. It aims to provide further information on the effectiveness of ICC-T, an interactive intervention designed for teachers to change their attitudes towards violence and to learn how to use non-violent discipline measures in school settings in a practical way. The evidence from the proposed cRCT will enable us to link violence reduction to education, health, and societal outcomes and thus provide valuable gateway for communities, the education system, governments and policy-makers to guide actions and investments in promoting the safety of school-going children and adolescents in Pakistan.

## Data Availability

During the project, anonymized data and materials will be shared only with partners working in other ICC-T projects and other researchers working in prevention of violence against children upon request. After the completion of the project, the anonymized data and materials will be available to the public.
